# Effectiveness of IT-based diabetes management interventions: a review of the literature

**DOI:** 10.1186/1471-2296-10-72

**Published:** 2009-11-17

**Authors:** Beth M Costa, Kristine J Fitzgerald, Kay M Jones, Trisha Dunning AM

**Affiliations:** 1 School of Nursing, Faculty of Health, Medicine, Nursing and Behavioural Sciences, Deakin University, Waterfront Campus, Geelong, 3220, Victoria, Australia; 2 Department of General Practice, Monash University, Notting Hill, 3168, Victoria, Australia; 3 Barwon Health, Kitchener House, PO Box 281, The Geelong Hospital, Geelong Victoria, 3220, Australia

## Abstract

**Background:**

Information technology (IT) is increasingly being used in general practice to manage health care including type 2 diabetes. However, there is conflicting evidence about whether IT improves diabetes outcomes. This review of the literature about IT-based diabetes management interventions explores whether methodological issues such as sample characteristics, outcome measures, and mechanisms causing change in the outcome measures could explain some of the inconsistent findings evident in IT-based diabetes management studies.

**Methods:**

Databases were searched using terms related to IT and diabetes management. Articles eligible for review evaluated an IT-based diabetes management intervention in general practice and were published between 1999 and 2009 inclusive in English. Studies that did not include outcome measures were excluded.

**Results:**

Four hundred and twenty-five articles were identified, sixteen met the inclusion criteria: eleven GP focussed and five patient focused interventions were evaluated. Nine were RCTs, five non-randomised control trials, and two single-sample before and after designs. Important sample characteristics such as diabetes type, familiarity with IT, and baseline diabetes knowledge were not addressed in any of the studies reviewed. All studies used HbA_1c _as a primary outcome measure, and nine reported a significant improvement in mean HbA_1c _over the study period; only two studies reported the HbA_1c _assay method. Five studies measured diabetes medications and two measured psychological outcomes. Patient lifestyle variables were not included in any of the studies reviewed. IT was the intervention method considered to effect changes in the outcome measures. Only two studies mentioned alternative possible causal mechanisms.

**Conclusion:**

Several limitations could affect the outcomes of IT-based diabetes management interventions to an unknown degree. These limitations make it difficult to attribute changes solely to such interventions.

## Background

Recent advances in Information Technology (IT) have been used to manage chronic diseases including diabetes mellitus. Specifically, IT can enhance communication among health professionals (HPs) and patients [1], and improve diabetes management [2]. A number of IT applications are currently available including electronic patient registers, electronic decision support systems, videoconferencing, telemedicine, biometric devices capable of uploading information such as blood glucose test results to the Internet or the HP's computer and Internet-based interactive patient support networks [3].

Type 2 diabetes mellitus is a chronic progressive disease associated with long term complications and high morbidity and mortality rates [4]. Between 1999 and 2000 the Australian prevalence of diabetes was 7.4% [5] and is projected to reach 15% by 2031 [6]. Type 2 diabetes accounts for 85% to 96% of all diagnosed cases and is reported to be largely due to lifestyle factors such as calorie dense diets, obesity and inactivity [5].

Diabetes management includes achieving HbA_1c _< 7%, reducing hyperglycaemia symptoms, preventing complications, appropriate self-care, and enhancing quality of life [7,8]. In Australia, general practitioners (GPs) receive financial incentives to use structured GP management plans (GPMPs) and work collaboratively with nurses and allied HPs in Team Care Arrangements (TCAs) to manage a number of chronic diseases including diabetes. Interdisciplinary TCAs can result in diabetes care consistent with management guidelines and HbA_1c _close to 7% [9]. However, diabetes management is often inadequate despite care plans [10].

Several barriers prevent GPs in Australia from using structured diabetes care plans (Table 1). Many of these barriers relate to insufficient time, knowledge and inadequate communication among HPs. A shift in focus from acute to chronic care is needed to successfully address these barriers. A structured approach to care planning, which could be facilitated by IT, could begin to address barriers associated with time constraints, knowledge deficits and inadequate communication among HPs. Significantly, IT care planning systems need to be easily integrated into general practice. However, there appears to be little research conducted in Australia that has examined the impact of IT on diabetes management.

**Table 1:** Barriers to using diabetes interventions in general practice identified in the literature.

Intervention	Barrier
Care plans	Time constraints
	Amount of documentation required to claim *EPC items
	Inconsistency between chronic disease care and acute care-oriented systems
	Conflicting patient and ^+^GP care goals
	Inadequate knowledge about available ^#^AH services
	Inadequate training to work within interdisciplinary teams
	Difficulty communicating with other °HPs
	Long waiting lists
	No practice nurses employed within the practice
Electronic templates	Time constraints
	Unsure of process
	Perception that the items were too business-focused
^α ^IT-based diabetes interventions	Concerns about confidentiality
	Staff shortages
	Time constraints
	Inadequate training
	Anxiety about change

Note. *EPC = Enhanced Primary Care; ^+^GP = general practitioner; ^#^AH = Allied Health; °health professionals; ^α^IT = information technology.

One Australian study explored GP's and HP's use of computer-based GPMP and TCA templates to plan diabetes care [11]. GPs and HPs had both positive and negative views about the templates (Table 1). One reason could be that electronic templates have not addressed many of the barriers to using care plans shown in Table 1. The authors concluded that, while GPs used the templates to claim Medicare items, most did not feel care plans improved the overall care of patients with chronic diseases [11].

A recent literature review examined 29 IT-based diabetes management intervention studies using electronic medical records (EMR) and undefined web-based programs, within general practice [12]. Several benefits of IT-based interventions were discussed and a number of barriers were identified, including some of those shown in Table 1.

The review found the frequency with which diabetes-specific investigations were performed increased following the introduction of IT-based interventions [12]. However, improved HbA_1c _and cholesterol levels were inconsistent among the studies reviewed. The review did not scrutinise the study methodologies. It is possible that methodological factors could have influenced the results, and need to be taken into account to determine whether they could help explain the inconsistent findings. Thus, using IT in diabetes management does not appear to have overcome existing IT management barriers in general practice, especially time constraints and inadequate training.

The aim of the current review of the literature about IT-based diabetes management interventions was to determine the extent to which methodological issues could explain the inconsistent findings among studies that examined the effect of IT-based management interventions on diabetes outcomes. The review was undertaken to answer the following question: what are the methodological limitations associated with (1) sample characteristics, (2) outcome measures and (3) mechanisms causing change in the outcome measures of studies that evaluated IT-based diabetes management interventions in general practice? Cost-benefit and cost-effectiveness issues are not addressed in this paper.

## Methods

### Search Strategy

Databases searched included the Medical Literature Analysis and Retrieval System Online (Medline), the Cumulative Index to Nursing and Allied Health Literature (CINHAL), PubMed, and the American Psychological Association Online Database (PsychInfo). The reference lists of extracted articles were hand searched for additional relevant articles. Search terms using the Medical Subject Headings (MeSH) thesaurus were: diabetes mellitus, type 2/or diabetes mellitus AND disease management AND informatics OR computerised medical records systems.

### Inclusion Criteria

Original articles that evaluated an IT diabetes management intervention in general practice published between 1999 and 2009 inclusive in English were reviewed. These included randomised control trials (RCTs) and pretest-post-test designs.

### Exclusion Criteria

Articles that evaluated IT diabetes management interventions in hospital settings or other chronic diseases were excluded. Articles that did not include diabetes-related metabolic outcomes (HbA_1c_, lipids, renal function) as a primary outcome measure, literature review papers, and opinion articles were also excluded.

### Data Analysis

Titles and abstracts were independently reviewed by two of the authors during January and February 2009. Following the initial abstract review, complete articles were retrieved if they met the inclusion criteria. The Australian National Health and Medical Research Council (NHMRC) guidelines for appraising the quality of intervention studies [13] were used to assess the studies reviewed. Where the eligibility of an article was unclear, the authors engaged in discussion until consensus was reached. A systematic review of the relevant studies was not conducted because the study designs and evaluated interventions were heterogeneous. Therefore, guidelines for undertaking systematic reviews such as the PRISMA statement [14] were not followed.

## Results

### Descriptive Statistics

A total of 425 articles were identified. Sixteen met the inclusion criteria [15-30] (Table 2). Two articles were based on a single intervention as part of the American Longitudinal Informatics for Diabetes Education and Telemedicine (IDEATel) Project, and separately examined psychological [19] and physiological [18] outcomes. The remaining 14 articles evaluated 14 different IT-based interventions. Fourteen studies were conducted in the United States of America, one in the United Kingdom, and one in Korea. No Australian studies met the inclusion criteria. Eleven studies evaluated GP-targeted interventions including: diabetes registries [16,17,25,27,29,30], electronic management systems [15,24,26,28] and EMRs [21]. Five studies evaluated patient-targeted interventions including: telemedicine [18,19], web-based electronic self-management systems [22,23] and electronic software support programs [20].

**Table 2:** Summary of the 16 Studies Reviewed

Design	Intervention	Country	Focus	Sample	Sample size (n)	Duration	Reference
Cluster blind RCT	Electronic decision support system	USA	GP	T1D, T2D	639	21 months	[15]
Cluster RCT	Diabetes register	UK	GP	T2D	3, 608	15 months	[16]
Cluster RCT	Diabetes register	USA	GP	T2D	7, 101	12 months	[17]
Cluster RCT	Electronic telemedicine	USA	Patient	T2D	1, 665	12 months	[18]
Cluster RCT	Electronic telemedicine	USA	GP	T2D	1, 665	12 months	[19]
Cluster RCT	Electronic self-management system	USA	Patient	T2D	886	12 months	[20]
RCT	Electronic self-management system	USA	Patient	T1D, T2D	122	6 months	[21]
RCT	Electronic self-management system	USA	Patient	T1D, T2D	104	12 months	[22]
RCT	Electronic self-management system	USA	Patient	T1D, T2D	62	6 months	[23]
Nonrandomised cluster control trial	Electronic management system	USA	GP	T1D, T2D	6, 646	24 months	[24]
Nonrandomised control trial	Diabetes register	USA	GP	T1D, T2D	898	20 months	[25]
Nonrandomised control trial	Personal Digital Assistant (PDA) based electronic management system	USA	GP	T2D	173	16 months	[26]
Nonrandomised control trial	Diabetes register	USA	GP	T1D, T2D	661	2 years	[27]
Nonrandomised control trial	Electronic decision support system	USA	GP	T1D, T2D	16	6 months	[28]
Single sample before and after design	Diabetes register	USA	GP	T1D, T2D	62	3 years	[29]
Single sample before and after design	Diabetes register	Korea	GP	T1D, T2D	185	3 months	[30]

Note. RCT = randomised control trial; GP = general practitioner; T1D = type 1 diabetes; T2D = type 2 diabetes.

Nine studies were RCTs; three involved randomisation by GP clusters and two by patients. Only one RCT indicated that blinding procedures were used. Five studies were non-randomised control trials. Two studies used a single sample before and after design.

Limitations were reported in all the studies reviewed (Table 3). Reported limitations included study design, sample characteristics, unmeasured variables confounding outcome measures, and participant attrition. The following section describes the limitations of the studies included in the review according to three issues: sample characteristics, outcome measures, and mechanisms causing change in the outcome measures.

**Table 3:** Limitations associated with study outcomes in 16 studies reviewed

Issue	Limitation
Design	Not randomised [24,27]
	No control group [28]
	Short duration of the intervention [[15], 1623]
Sample	Sample not representative [22,23]
	Small sample size [28]
	Sampling population restricted to patients who accessed hospital web page [30]
Intervention effect	Unable to determine intervention component contributing to the observed change [23,29]
	Unmeasured variable contributing to observed change [28]
	Possibility of increased attention to management contributing to observed change [19,20]
	Mean baseline HbA_1c _close to 7% [17,20,25]
	Concomitant exposure to another intervention [27]
Participant dropout	Non-random participant dropout [19]
	Complete data not available for all participants [29]

### Sample Characteristics

Nine studies included both type one and type two diabetes patients, two of these evaluated patient-focused interventions [22,23]. None of the studies examined the effect of diabetes type on the outcome measures.

The IT-based interventions had varying degrees of IT complexity. Yet patient and GP baseline levels of familiarity and confidence in their ability to use computers were not reported. Diabetes knowledge at baseline was not reported in any of the studies reviewed, even though a main component of patient-focused interventions consisted of providing self-management education.

### Outcome measures

All of the studies reviewed included HbA_1c _as a primary outcome. One study included HbA_1c _as the only outcome measure [29]. Nine studies reported a significant reduction in mean HbA_1c _at three months [30], six months [23,28], 12 months [17-19], three years [29] and five years [21]. Of these, one RCT [22] and one non-randomised control trial [21] reported significantly lower mean HbA_1c _in the intervention groups compared to controls at follow up (*p *< 0.05). One study reported that minimal use of diabetes registers was associated with an increase in mean HbA_1c _from 7.4 at baseline to 7.8 at follow up (*p *< .05) [27]. Two of the studies reported the HbA_1c _assay method [21,22]. A change in the HbA_1c _assay method during one study led to significantly lower HbA_1c _concentrations [21].

Mean baseline HbA_1c _in eight of the studies reviewed ranged between 7.0 and 7.3% [15,17-20,23,24,26]. Interventions introduced when the mean baseline HbA_1c _was 9.0 - 11.0% were associated with a significant improvement in HbA_1c _(7.5 - 7.68%).

Cholesterol levels were assessed in all the studies reviewed except one [29]. Ten studies measured blood pressure [15-19,22-24,26,28], two studies measured weight [23,28], and one measured creatinine [16]. Eight assessed GP adherence to diabetes complication screening guidelines; five assessed whether diabetes-specific metabolic indicators were documented in patient files [16,17,29,21,25] and six studies assessed whether annual foot and eye tests were performed [16,17,20,24,26,27]. GP adherence measures assessed could not be found in one study [28].

Five studies reported prescribed oral hypoglycaemic agents (OHAs) and insulin [15,16,22,25,28]. Four of these measured the change in the proportion of participants prescribed OHAs and insulin at baseline and follow up [15,16,25,28]. The number of patients that were prescribed these medicines increased over a 21 month period.

Two studies reported psychological outcomes; specifically self-efficacy and depression. One study reported increased self-efficacy using the Diabetes Self-Efficacy Scale (DSES) [31] following the introduction of a patient-targeted IT intervention [19]. A second study reported the prevalence of depression reduced from 19% to 12% in the intervention group and 16% to 13% in the comparison group in a 12-month evaluation of a patient-directed electronic assessment and action plan software program [20]. Patient lifestyle factors were not reported in any of the studies reviewed.

### Mechanisms Causing Change in the Outcome Measures

The causal mechanism responsible for changes in the outcome measures in the studies reviewed was an IT-based intervention. Three studies acknowledged that increased attention to diabetes care or some unmeasured factor could have contributed to the changes observed in outcome measures [15,25,30].

## Discussion

Nine of the studies reviewed identified an association between the IT intervention used and the outcome measures [17-19,21-23,28-30]. However, caution should be exercised in interpreting the findings of studies, which evaluated IT-based diabetes management interventions because a number of different confounding factors may have influenced the outcomes, some of which were considered in the current paper.

### Sample Characteristics

In IT-based diabetes intervention evaluation studies, sample characteristics such as diabetes type, familiarity with IT, and baseline diabetes-related knowledge and skills may be important factors that could influence the results. None of the studies appeared to address any of these factors. While RCT randomisation procedures aim to reduce the effect of known and unknown confounding variables [32] it may not be possible to eliminate their effect completely. Furthermore, randomisation was not undertaken in seven of the studies reviewed [24-30]. A lack of randomisation may have increased the risk of bias when assigning participants to intervention and control groups. This may have influenced the outcome measures to an unknown degree.

#### Diabetes Type

Type one diabetes is unique in that management always involves medication, whereas type two diabetes may be managed using diet and lifestyle. These differences could influence individuals' self-management strategies and affect outcome measures.

#### Familiarity with IT

Cognitive ability affects engagement with technology such as learning new skills and new ways of performing familiar tasks such as emailing, participating in chat rooms and navigating the Internet [33]. Advanced disease progression, multiple co-morbidities, and natural ageing, may reduce the cognitive capacity of participants to learn the skills necessary to engage with IT-based interventions. Thus, cognitive ability could have influenced the outcome measures of some studies to an unknown degree, but was not discussed in the studies reviewed.

Type 2 diabetes is common in older adults (> 60 years) [34]. Compared with younger adults, older adults report greater anxiety about using computers, lower use of technology [33] and less confidence in their ability to use computers [35]. Therefore, some patients may have experienced anxiety about engaging in computer-related interventions such as accessing support materials on the Internet and uploading blood glucose test results to study websites. However, these potential confounders were not explored or discussed in any of the studies reviewed.

GPs' initial level of computer literacy and experience with the technology may influence their acceptance and use of new informatics-based interventions [36,37]. Some GPs who trained before the introduction of computers into general practice may be less confident to use them than other GPs, such as more recently trained GPs. High levels of computer anxiety may be associated with reluctance to engage with online interventions to an unknown degree. Therefore, when evaluating any new IT intervention in general practice it is important to examine professionals' perceptions and acceptance of the technology, because these factors could influence the outcomes of the intervention, yet this was not assessed in any of the studies reviewed.

#### Diabetes Knowledge

Not all patients with diabetes will benefit from an IT-based management intervention [1]. Many individuals, particularly those diagnosed many years prior to participating in an intervention study, may have already developed strategies and acquired the relevant knowledge to manage their diabetes with minimal professional intervention. In particular, patients who are able to maintain their HbA_1c _close to 7% may have well developed self-management strategies and knowledge. Self-management education interventions may only be effective for participants with inadequate diabetes-specific knowledge. Therefore, it may be of value to assess diabetes knowledge and skill levels prior to introducing interventions that aim to up-skill individuals with diabetes. This was not reported in any of the studies reviewed.

### Outcome measures

#### Diabetes Management Goals

HbA_1c _was the primary outcome measure used in the studies reviewed. HbA_1c _provides an average measure of blood glucose over the previous 90 to 120 days [8]. It is an objective measure of glycaemic control and is unaffected by self-report bias. However, a number of factors can affect HbA_1c _and thus influence the outcome. First, frequent hypoglycaemic episodes, anaemia, haemoglobinopethies and excessive blood loss can lower HbA_1c _[7]. These factors were not assessed in any of the studies reviewed. Likewise, they are rarely, if ever, considered when developing care plans.

Second, HbA_1c _may be an appropriate outcome measure only when it is elevated at baseline. Individuals with good diabetes self-management before participating in an IT-based intervention may not derive any benefit from participation and may confound study results. IT diabetes management interventions may need to be introduced to populations where diabetes is poorly controlled in order to effect changes, as was the case in three of the studies reported [22,28,29].

Third, there may be interlaboratory variations if HbA_1c _was not measured in the same laboratory. Thus, it may be unwise to make comparisons among studies using different laboratories for HbA_1c _results. As well as process and structure outcomes, psychological state, medication status and lifestyle factors need to be assessed.

#### Diabetes Medications

When lifestyle changes alone do not control blood glucose levels among people with type 2 diabetes, glucose lowering medications (OHAs or insulin) are introduced [8]. Medications, doses, and dose intervals are altered over time as needed to maintain metabolic control. Therefore, medication status at baseline and changes over time can provide an indication of disease progression in type 2 diabetes. None of the studies reviewed assessed the effect of OHAs or insulin on outcomes or whether the medicine regimens changed because of the IT interventions. Without such information, it is not possible to tease out the effects of OHAs or insulin on outcomes or to attribute change solely to IT-based interventions.

#### Psychological Factors

Psychological factors were rarely measured in any of the studies reviewed. However, a range of psychological variables can influence glycaemic control. High levels of diabetes self-efficacy are directly associated with lower HbA_1c _[38,39], yet only one study measured changes in self-efficacy following the introduction of an IT-based intervention [19]. One study evaluated a patient-directed IT intervention that used electronically delivered strategies to enhance diabetes self-efficacy beliefs and self-management behaviours, yet did not report self-efficacy as an outcome measure [23]. While the authors reported an improvement in HbA_1c _following the intervention, it is not clear whether the change was due to a change in participants' self-efficacy, the intervention, or any other factor. Thus, caution must be exercised when attributing the findings of this study to the IT intervention.

Depression is common among people with diabetes and is associated with poor metabolic control, poor treatment adherence and reduced quality of life [5], and may influence outcomes of studies evaluating new diabetes management interventions. Yet depression was only considered in one of the studies reviewed [20]. Furthermore, there is only limited evidence that IT-based interventions reduce depression, therefore the variable causing a change in depression levels in Glasgow et al.'s study [20], is unclear. Being part of a study or receiving increased attention and care from HPs may reduce depression among study participants rather than the intervention.

#### Lifestyle Factors

Social and lifestyle factors influence how well people manage their diabetes. Income and education deficits are associated with inadequate self-care, and affect diabetes outcomes [5]. Inadequate diabetes-related knowledge and skills impair people's ability to successfully undertake self-management [40]. However, lifestyle factors were not reported in any of the studies reviewed, and represents a significant limitation of the current literature.

### Mechanisms Causing Change in the Outcome Measures

The first possible mechanism responsible for reported changes in outcome measures, the mechanism considered in all of the studies reviewed, was the IT mode of delivery. That is, delivering diabetes management interventions electronically is a possible mechanism causing change in the outcome measures. However, factors other than the method of delivery could contribute to observed changes in outcome measures.

Good diabetes management and GP adherence to management guidelines may partially account for changes in HbA_1c_. Most of the studies reviewed compared the IT-based intervention to standard diabetes management. Appropriate diabetes management effects a change in outcome measures, regardless of the mode of delivery. Yet the appropriateness of people's management regimens was not discussed. It is unethical to withhold treatment with proven efficacy in order to provide a control group for IT interventions. However, the possibility that diabetes management per se and not the IT method influenced outcomes should be considered.

Participating in research can influence outcome measures. Individuals who choose to take part in a research study may be motivated to perform well to help the researcher and confirm to themselves their contribution is valuable [41]. Therefore, participating in an IT-based intervention study may have influenced self-management behaviours in both the intervention and comparison groups, and in turn affected the outcomes, but this possibility was not acknowledged in any of the studies reviewed.

### Limitations

There are several limitations to the current review, which could affect the conclusions. First, a systematic literature review was not undertaken because of the heterogeneity of the interventions and study designs of the literature identified. Second, while a search strategy was used, the review may have omitted important studies evaluating other IT-based diabetes management interventions. Finally, the review did not identify any studies conducted within Australia. The Australian health system is unique to the health systems of both the United Kingdom and the United States, where the majority of the studies reviewed were conducted. The availability of financial incentives for using structured care planning within Australia may influence GPs' use of IT-based diabetes management interventions to an unknown degree.

## Conclusion

Given the growing diabetes epidemic, effective diabetes management interventions suited to general practice are needed. Due to the limitations of the studies reviewed, the effectiveness of current IT-based interventions is unclear and difficult to attribute solely to the interventions. Future research efforts must give thoughtful attention to methodological issues to produce valid, reliable and generalisable findings. In particular, possible confounding factors need to be acknowledged and controlled, outcome measures need to be relevant to the populations being studied and appropriate methods used to address study aims.

## Competing interests

The authors declare that they have no competing interests.

## Authors' contributions

BC and KF performed the literature review. BC drafted the manuscript with KF. KJ and TD helped identify relevant papers and commented on successive drafts of the paper. TD conceived the paper and participated in its design. All the authors read and approved the final manuscript.

## Pre-publication history

The pre-publication history for this paper can be accessed here:

http://www.biomedcentral.com/1471-2296/10/72/prepub

## References

[B1] Piette JD Interactive behaviour change technology to support diabetes self-management: where do we stand? Diabetes Care 2007 30 2425-32 10.2337/dc07-1046 17586735

[B2] Joshy G Simmons D Diabetes information systems: a rapidly emerging support for diabetes surveillance and care Diabetes Technol Ther 2006 8 587 597 10.1089/dia.2006.8.587 17037973

[B3] Nobel J Bridging the knowledge-action gap in diabetes: information technologies, physician incentives and consumer incentives converge Chronic Illn 2005 2 59 69 10.1177/17423953060020010201 17175683

[B4] Department of Human Services Diabetes prevention and management: a strategic framework for Victoria 2007-2010 2007 Melbourne: Victorian DOH

[B5] Australian Institute of Health and Welfare Diabetes: Australian facts 2008 Diabetes series no. 8 Cat. No. CVD 40 edition 2008 Canberra: AIHW

[B6] Queensland Health The health of Queenslanders 2008: prevention of chronic disease. Second report of the Chief Health officer Queensland 2008 Brisbane: QLD Health

[B7] Dunning T Care of people with diabetes: a manual of nursing practice 2003 2 Carlton South: Blackwell Publishing

[B8] Diabetes Australia and Royal Australian College of General Practitioners Diabetes management in general practice: guidelines for type 2 diabetes 2008 14 Norah Head, NSW: Diabetes Australia Publication

[B9] Zwar NA Hermiz O Comino EJ Shortus T Burns J Harris M Do multidisciplinary care plans result in better care for patients with type 2 diabetes? Aust Fam Physician 2007 36 85 9 17252093

[B10] Harris M Challenges in diabetes management Aust Fam Physician 2008 37 716-20 18797529

[B11] Bolger-Harris H Schatner P Saunders M Using computer based templates for chronic disease management Aust Fam Physician 2008 37 285 288 18398531

[B12] Adaji A Schatner P Jones K The use of information technology to enhance diabetes management in primary care: a literature review Inform Prim Care 2008 16 229 237 19094410 10.14236/jhi.v16i3.698

[B13] National Health and Medical Research Council How to review the evidence: systematic identification and review of the scientific literature 2000 Canberra: NHMRC

[B14] Liberati A Altman DG Tetzlaff J Mulrow C Gotzsche PC Ioannidis JPA Clarke M Devereaux PJ Kleljnen J Moher D The PRISMA statement for reporting systematic reviews and meta-analyses of studies that evaluate healthcare interventions: explanation and elaboration BMJ 2009 339 b2700 10.1136/bmj.b2700 19622552 PMC2714672

[B15] Smith SA Shah ND Bryant SC Christianson TJH Bjornsen SS Giesler PD Chronic care model and shared care in diabetes: randomized trial of an electronic decision support system Mayo Clin Proc 2008 83 747 757 10.4065/83.7.747 18613991

[B16] Eccles MP Whitt PM Speed C Steen IN Vanoli A Hawthorne GC A pragmatic cluster randomised controlled trial of a Diabetes Recall and Management system: the DREAM trial Implementation Science 2007 2 6 12 17306017 10.1186/1748-5908-2-6 PMC1804280

[B17] Peterson KA Radosevic DM O'Connor PJ Nyman JA Prineas JR Smith SA Improving diabetes care in practice: Findings from the TRANSLATE trial Diabetes Care 2008 31 2238-2243 18809622 10.2337/dc08-2034 PMC2584171

[B18] Shea S Weinstock RS Starren J Teresi J Palmas W Field L A randomized trial comparing telemedicine case management with usual care in older, ethnically diverse, medically underserved patients with diabetes mellitus J Am Inform Assoc 2006 13 40 51 10.1197/jamia.M1917 PMC1380195 16221935

[B19] Trief PM Teresi JA Eimicke JP Shea S Weinstock RS Improvement in diabetes self-efficacy and glycaemic control using telemedicine in a sample of older, ethnically diverse individuals who have diabetes: the IDEATel project Age Ageing 2009 38 2 219 225 10.1093/ageing/afn299 19171951

[B20] Glasgow RE Nitting PA King DK Nelson CC Cutter G Gaglio B Randomized effectiveness trial of a computer-assisted intervention to improve diabetes care Diabetes Care 2005 28 33 39 10.2337/diacare.28.1.33 15616230

[B21] O'Connor PJ Crain AL Rush WA Sperl-Hillen JM Gutenkauf JJ Duncan JE Impact of an electronic medical record on diabetes quality of care Ann Fam Med 2005 3 300 306 10.1370/afm.327 16046561 PMC1466905

[B22] McMahon GT Hu TM Gomes HE Levine BA Hohne SH Conlin PR Web-based care management in patients with poorly controlled diabetes Diabetes Care 2005 28 1624 1629 10.2337/diacare.28.7.1624 15983311 PMC1262644

[B23] Bond GE Burr R Wolf FM Price M McCurry SM Teri L The effects of a web-based intervention on the physical outcomes associated with diabetes among adults ag 60 and older: a randomized trial Diabetes Technol Ther 2007 9 52 59 10.1089/dia.2006.0057 17316098 PMC2748064

[B24] Montori VM Dinneen SF Gorman CA Zimmerman BR Rizza RA Bjornsen SS The impact of planned care and a diabetes electronic management system on community-based diabetes care Diabetes Care 2002 25 1952 1957 10.2337/diacare.25.11.1952 12401738

[B25] Grant RW Cagliero E Sullivan CM Dubey AK Estey GA Weil EM A controlled trial of population management: Diabetes Mellitus: putting evidence into practice (DM-PEP) Diabetes Care 2004 27 2299 2304 10.2337/diacare.27.10.2299 15451891

[B26] Jones D Curry W Impact of a PDA-based diabetes electronic management system in a primary care office Am J Med Qual 2006 21 401-7 17077422 10.1177/1062860606293594

[B27] Pollard C Bailey KA Petitte T Baus A Swim M Hendryx M Electronic patient regisries improve diabetes care and clinical outcomes in rural community health centers J Rural Health 2009 25 77 84 10.1111/j.1748-0361.2009.00202.x 19166565

[B28] Smith KE Levine BA Clement SC Hu M Alaoui A Mun SK Impact of MyCareTeam for poorly controlled diabetes mellitus Diabetes Technol Ther 2004 6 828 835 10.1089/dia.2004.6.828 15684636

[B29] Chima CS Farmer-Dziak N Cardwell P Snow S Use of technology to track program outcomes in a diabetes self-management program J Am Diet Assoc 2005 105 1933 1938 10.1016/j.jada.2005.07.013 16321600

[B30] Kwon H Cho J Kim H Lee J Song B Oh J Development of web-based diabetic patient management system using short message service (SMS) Diabetes Res Clin Pract 2004 66S S133 137 10.1016/j.diabres.2003.10.028 15563964

[B31] Shortridge-Baggett LM Bijl JJ Van Der International collaborative research on management of self-efficacy in diabetes mellitus J NY State Nurses Assoc 1996 27 9 14 9060718

[B32] Wang D Bakhai A (Eds) Clinical trials: a practical guide to design, analysis, and reporting. Chicago: Remedica 2006

[B33] Czaja SJ Charness N Fisk AD Hertzog C Nair SN Rogers WA Factors predicting the use of technology: findings from the center for research and education on aging and technology enhancement (CREATE) Psychol Aging 2006 21 333 352 10.1037/0882-7974.21.2.333 16768579 PMC1524856

[B34] Department of Planning and Community Development Ageing in Victoria discussion paper 2008 DPCD; Australia

[B35] Tacken M Marcellini F Mollenkopf H Ruoppila I Szeman Z Use and acceptance of new technology by older people: findings of the international MOBILATE survey "Enhancing Mobility in Later Life" Gerotechnology 2005 3 126 137 10.4017/gt.2005.03.03.002.00

[B36] Ludwick DA Doucette J Primary care physicians' experience with electronic medical records: barriers to implementation in a fee-for-service environment Int J Telemedicine and Applications 2009 Article ID 853524, 853529 10.1155/2009/853524 PMC2593889 19081787

[B37] Terry AL Thorpe CF Giles G Brown JB Harris SB Reid GJ Thind A Stewart M Implementing electronic health records: key factors in primary care Canadian Family Physician 2008 54 730 736 18474707 PMC2377228

[B38] Rose M Fliege H Hildebrandt M Schrop T Klapp BF The network of psychological variables in patients with diabetes and their importance for quality of life and metabolic control Diabetes Care 2002 25 35 42 10.2337/diacare.25.1.35 11772898

[B39] Williams GC McGregor HA King DK Nelson CC Glasgow RE Variation in perceived competence, glycemic control, and patient satisfaction: relationship to autonomy support from physicians Patient Educ Couns 2005 57 39 45 10.1016/j.pec.2004.04.001 15797151

[B40] Oldroyd J Proudfoot J Infante FA Davies GP Bubner T Holton C Providing healthcare for people with chronic illness: the views of Australian GPs Med J Aust 2003 179 30 33 12831381 10.5694/j.1326-5377.2003.tb05414.x

[B41] Nichols AL Maner JK The good-subject: investigating participant demand characteristics J Gen Psychol 2008 135 151 165 10.3200/GENP.135.2.151-166 18507315

